# Transdermal delivery of isoniazid and rifampin in guinea pigs by electro-phonophoresis

**DOI:** 10.1080/10717544.2016.1267275

**Published:** 2017-02-09

**Authors:** Suting Chen, Yi Han, Daping Yu, Fengmin Huo, Fen Wang, Yunxu Li, Lingling Dong, Zhidong Liu, Hairong Huang

**Affiliations:** 1 National Clinical Laboratory on Tuberculosis, Beijing Key Laboratory on Drug-Resistant Tuberculosis Research, Beijing Chest Hospital, Capital Medical University, Beijing Tuberculosis and Thoracic Tumor Institute, Beijing, China and; 2 Second Department of Thoracic Surgery, Beijing Chest Hospital, Capital Medical University, Beijing Tuberculosis and Thoracic Tumor Institute, Beijing, China

**Keywords:** Isoniazid, rifampin, transdermal delivery, electro-phonophoresis, guinea pigs

## Abstract

Electro-phonophoresis (EP) has been used as a drug delivery approach in clinical fields. The objective of the present study is to evaluate the skin permeability of isoniazid and rifampin in guinea pigs by EP to provide reference basis for clinical applications of such transdermal delivery system in the treatment of patients with superficial tuberculosis. Isoniazid and rifampin solutions were delivered transdermally with or without EP in health guinea pigs for 0.5 h. Local skin and blood samples were collected serially at 0, 1/2, 1, 2, 4, 6 and 24 h after dosing. Drug concentrations in local skin and blood were evaluated by high-performance liquid chromatography. Isoniazid concentrations in local skin of guinea pigs receiving isoniazid through EP transdermal delivery were significantly higher than in animals receiving only isoniazid with transdermal patch. However, for rifampin, patches alone group presented almost uniform concentration versus time curve with that of EP group, and both groups had concentrations much higher than the therapeutic concentration of the drug over sustainable time. After EP transdermal delivery, the mean peak concentrations of isoniazid and rifampin in skin were 771.0 ± 163.4 μg/mL and 81.2 ± 17.3 μg/mL respectively. Neither isoniazid nor rifampin concentration in blood could be detected (below the lower detection limit of 1 μg/mL) at any time point. The present study showed that application of EP significantly enhanced INH penetration through skin in guinea pigs, while RIF patch alone obtained therapeutic concentration in local skin. Our work suggests several possible medication approaches for efficient treatment of superficial tuberculosis.

## Introduction

Transdermal drug delivery has been applied clinically as a substitute medication method for oral administration or injections (Prausnitz & Langer, 2008). The efficiency of this approach has been validated in many diseases during the past 30 years, including different tumors, hypertension, angina etc. (Prausnitz et al., 2004).

Electro-phonophoresis (EP) is a new transdermal drug delivery method that combines electroporation and ultrasound, which have been proved to be active enhancement methods for transdermal drug delivery. However, the working mechanisms of both of these methods are very different, for example, electroporation involves transient creation of aqueous pores in the phospholipid bilayers of cell membranes by electric pulses and thus enhances the skin permeability for multiple types of molecules with different lipophilicity and size (Prausnitz et al., 1993; Zorec et al., 2013). On the contrary, enhancement of skin permeability caused by ultrasound is due to cavitation induced disorganization of the stratum corneum lipid bilayers and the occurrence of convective transport (Polat et al., 2011). EP had been developed to improve the efficiency of transdermal drug delivery, the synergistic effect of electroporation and ultrasound has been well documented in elsewhere (Kost et al., 1996). Recently, we validated EP for tuberculosis (TB) treatment and observed effective enhancement in the skin permeability of isoniazid (INH) and rifampin (RIF) in patients with tuberculous lymphadenitis. However, due to the invasive sampling method and ethical principles, evaluating EP systematically in humans was impossible in this assay.

In the current study, we aimed to examine the efficiency of EP in facilitating the INH and RIF penetration through skin of guinea pigs. The application of EP in the transdermal delivery of anti-TB drugs using guinea pigs as animal model has not been reported previously. Both isoniazid and rifampin belong to first-line anti-TB drugs and play key roles in TB treatment. INH is a hydrophilic drug; it can inhibit the mycobacterial cell wall construction whereas RIF, that is, a semisynthetic antibiotic from rifamycin B, is a lipophilic drug which can kill mycobacteria by inhibition of DNA transcription (Maggi et al., 1966). We chose those two most potent anti-TB drugs to speculate the role of EP for the treatment of TB lesion approximated to body surface.

## Material and methods

### Animals and drugs

Male guinea pigs weighing 300 ± 50 g were purchased from the Institute of Laboratory Animal Sciences, Chinese Academy of Medical Sciences and Peking Union Medical College. They were housed under standard conditions and fed a commercial mouse chow and water in the SPF animal laboratory of Beijing Tuberculosis and Thoracic Tumor Research Institute. All animal studies were in accordance with the institutional guidelines approved by the Animal Ethics Committee of Beijing Tuberculosis and Thoracic Tumor Research Institue. INH injection solution (Tianjin Jin Yao Amino Acid Co., Ltd, China) and RIF injection solution (Shenyang Shuang Ding Pharmaceutical Co., Ltd, China) with a concentration of 50 and 60 mg/mL respectively, were employed to prepare drug patches for transdermal delivery.

### Sample preparation

Separate groups of three guinea pigs were given transdermal patch (each of a 4 × 4 cm single piece of cotton) immersed with a single dose of 100 mg INH injection solution or 100 mg RIF injection solution with or without EP for 30 min. EP was conducted using ultrasonic conductometric instrument (Beijing Noah Tongzhou Medical Technology Co. Ltd., Beijing, China). The setting parameters included 1 MHz and intensity of 75 mW/m^2^. After transdermal delivery, residual agents on the skin surface were washed with tap water prior to sampling. Skin or blood samples were collected 0, 1/2, 1, 2, 4, 6 and 24 h after transdermal administration. Specifically, skin sample (∼0.2 g, 1.0 × 1.0 cm) was cut out carefully from the local skin in which the antibiotic was delivered. Blood (∼0.2 mL) was collected serially from guinea pigs into heparinized tubes by the heart punctures at each time-point after antibiotic dosing. All the samples from skin or blood were frozen at −80 °C before analysis.

After weighing the skin sample, two volumes of saline was added and all the skin samples were homogenized in a FastPrep-24 Instrument (MP Biomedicals Europe) for 45 s at 4 m/s by MP Bio FASTPREP-24. Homogenates were then centrifuged, supernatant was separated and three volumes of methanol was added, into the supernatant separated from skin homogenates or the plasma separated from blood samples and mixed thoroughly. After centrifugation, supernatant was transferred to glass injection vials for HPLC analysis.

### Drug concentration detection by HPLC

Ten microliters of the supernatant from local skin tissues or blood samples was analyzed by HPLC (Agilent 1200, Palo Alto, CA) using Agilent ZORBAX SB-Aq column (4.6 mm × 100 mm, 5 μm) guarded by a ZORBAX SB-Aq column (2.1 mm × 12.5 mm, 5 μm) and detected by a UV2000 ultraviolet detector set at a wavelength of 264 nm for INH and 340 nm for RIF. For INH analysis, the mobile phase consisted of 0.02 M heptanesulfonic acid sodium salt: methanol: acetonitrile (78:17:5, v/v/v) and the column was kept at 25 °C. For RIF analysis, the mobile phase consisted of 0.05 M phosphate buffer: methanol (30:70, v/v) and the column was kept at 25 °C. Standard curves were constructed by dissolving known concentration of INH (1–100 μg/mL) or RIF (2–600 μg/mL) into blank skin homogenates or blank plasma and treating them similar to the experimental samples. Six different concentrations were used for developing a standard curve. The concentration of INH and RIF in each tested sample was calculated on the basis of standard curve. Both of the detection limits of INH and RIF in skin sample or blood sample were 1 μg/mL.

### Statistical analysis

Statistical analysis of the *in vivo* data obtained after the transdermal application of the patches was performed by one-way analysis of variance (ANOVA) using SigmaStat 3.5 (Systat Software Inc., San Jose, CA). A *p* value of  < 0.05 was considered statistically significant.

## Results

**Figure 1.**
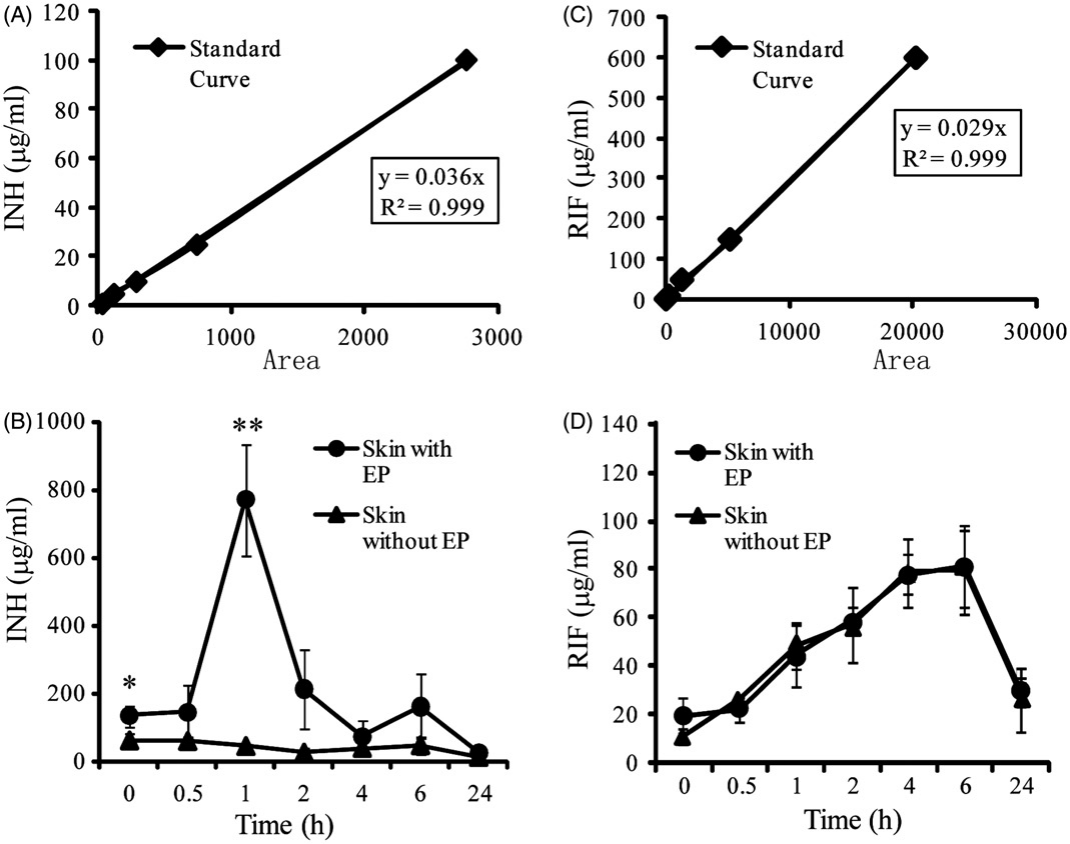
Local drug concentration in skin of guinea pigs receiving a transdermal patch with or without EP at different time points after dose administration. *p <0.05; **p< 0.01, significantly different from the control group that administered with transdermal patch alone.

The total running time of HPLC detection for INH and RIF was 15 and 10 min, respectively, while the retention time for INH and RIF were 6.23 ± 0.07 and 6.05 ± 0.04 min, respectively. The skin standard curves for INH and RIF ranged from 1 to 100 μg/mL and 2 to 600 μg/mL, respectively. The standard curve correlation coefficient for INH and RIF ranged between 0.996 and 0.999 in both methanol and extracted skin supernatant (Figure 1A and B). The absolute recoveries from skin were greater than 70%, that is, 72.5–76.5% for INH and 78.0–86.5% for RIF, respectively. The within-day precision (percentage coefficient of variation (CV)) of validation quality control (QC) samples (100, 25 and 5 μg/mL) for INH was 0.7–3.1% and that of intraday was 5.6–14.1%. The within-day precision (%CV) of validation QC samples (50, 10 and 2 μg/mL) for RIF was 0.6–6.2% and that of intra-day was 3.1–19.6% (Table 1). As a whole, the data above demonstrated that the method provided good linearity and high recoveries of the analytes, with sufﬁcient sensitivity, precision and accuracy for simultaneous measurement of INH and RIF in small size skin samples from hairless guinea pigs.

**Table 1.** Intra-/Interday assay precision and accuracy of the HPLC method for the measurement of INH and RIF in local skin of guinea pigs.

Intraday variation				Interday variation			
Concentration (μg/mL)	Mean concentration measured	% CV	% Accuracy	Concentration (μg/mL)	Mean concentration measured	% CV	% Accuracy
INH							
5	4.3	3.1	85.5	5	4.7	14.1	94.0
25	20.0	1.8	80.0	25	21.3	5.6	85.2
100	94.8	0.7	94.8	100	102.6	6.5	102.6
RIF							
2	2.2	6.2	110.0	2	2.7	19.6	135.0
10	9.5	0.6	95.0	10	9.9	3.3	99.0
50	48.7	0.6	97.4	50	50.4	3.1	100.8

Accuracy (%) = 100 × (mean concentration/nominal concentration).

CV (%) = 100 × (standard deviation/mean concentration).

For animals treated with INH patches from 0.5 to 1 h through EP, as shown in Figure 1(B), the increased trend of local INH concentration was most obvious. The highest level of INH in skin sample was found to be 771.0 ± 163.4 μg/mL at the 1-h time point. The local drug concentration decreased gradually until 24 h, and the INH concentration at the 24-h time point was 26.7 ± 12.4 μg/mL. Furthermore, when comparing concentrations at each time point for both routes of administration, INH concentrations in the local skin of animals dosed by the EP route were 2- to 17-fold higher than those observed in receiving INH patches alone animals, and statistically comparable INH concentrations (*p *<0.01) were observed at the 0- and 1-h time points.

Regarding RIF delivery, patches alone group presented almost uniform concentration-versus-time curve with that of EP group (Figure 1D). After dose administration, the level of RIF in local skin increased at 6 h. Compared to transdermal patches alone recipients, RIF absorbed faster when administered by the EP at initial time point (0 h), but the difference was not significant. In EP group, local drug concentration of RIF was 19.4 ± 7.4 μg/mL at 0-h time point. Local drug concentration at 6-h time point was highest, up to 81.2 ± 17.3 μg/mL and could still maintain a drug concentration of 29.1 ± 6.3 μg/mL at 24-h time point, significantly greater than the minimal inhibitory concentration (MIC) of RIF (0.25 μg/mL).

Drug concentrations in blood were too low to be detected by HPLC assay with UV2000 ultraviolet detector at any time point. Neither INH nor RIF was detected in the plasma samples in any of the treatment groups, reflecting the negligible circulation system absorption of the drugs by this localized drug delivery method.

## Discussion

The working principle of ultrasonic conductometric instrument involves inducing hole by electroporation and high-end physical means such as cavitation under ultrasonic condition, between the skin and cell membrane (Kost et al., 1996). Through these specific artificially formed channels, drug can directly reach the lesion organs and tissues. Furthermore, drugs rapidly spread in the local high infiltration area, promoting the uptake of drugs into the cytosol (Kost et al., 1996). It has the advantage of acting directly on the lesion tissue, reducing first-pass drug-degradation effects; promoting the transport of drugs into the cells, improving the bioavailability; reducing side effects for without going through the whole-body blood circulation. Meanwhile, it has a long time course efficacy, and is suitable for various drugs due to several advantages, for example, cross infection free and noninvasive, no pain involved and the ease to use (Prausnitz et al., 2004; Paudel et al., 2010). The clinical verification which shows the therapeutic doses of ultrasound are safe to human body has been certificated for many years (Polat et al., 2011). A series of experiments verified that ultrasonic penetration was effective for many drugs (Prausnitz & Langer, 2008; Polat et al., 2011). Using these novel second- and third-generation enhancement strategies, including microneedles, thermal ablation, microdermabrasion, electroporation and cavitational ultrasound, the transdermal delivery is poised to significantly increase its impact on medication (Prausnitz et al., 2004; Paudel et al., 2010; Wiedersberg & Guy, 2014).

In the present study, skin penetration enhancement of the two most commonly used anti-TB drugs by EP in a 24-h period was analyzed in healthy guinea pigs using ultrasonic conductometric instrument. We did not wash off the agents on the skin surface immediately after transdermal delivery but before sampling, and found that the concentration of INH in skin continued to rise in an hour after the transdermal delivery by EP and reached its highest level at 1-h time point; while the RIF was absorbed more slowly and the highest concentration was found at 6-h time point. Furthermore, the local skin absorption of RIF which is a lipophilic drug with EP was similar to that without EP, whereas that for hydrophilic drug INH with EP was increased 2–17-fold. Thus, EP has a great effect on the skin permeation of INH which is a hydrophilic drug and usually has low permeability. This result is in agreement with a previous study by H Ueda et al. (Ueda et al., 1995) showing that skin permeability of hydrophilic drugs increased by ultrasound while that of lipophilic drugs slightly increased. On the other hand, our work demonstrated that patches alone with RIF can obtain higher than therapeutic drug concentration in the local skin, which is very promising for superficial tuberculosis treatment because of being easy, simple and cheap.

However, these results are a little different from that of our previous study, in which we examined whether EP can effectively increase the penetration of the INH and RIF through skin in tuberculous lymphadenitis patients and verified that application of EP significantly enhances both INH and RIF penetration through skin (Chen et al., 2016). Because of the difficulty of obtaining the pyogenic fluids, the pharmacokinetic studies of the change of drug concentrations in pyogenic fluids over an extended period of time and the optimal ultrasonic intensity and dosage were not addressed in previous study. In the present study, we use healthy guinea pigs as animal model to find the enhancement of the INH and RIF permeation through skin within 24 h after transdermal delivery with EP or without EP. Compared with the previous study researched in tuberculous lymphadenitis patients, here, we found that the enhancement of the RIF permeation in guinea pigs’ skin is not obvious by EP, while significant RIF concentration was achieved in the local lesion and its surroundings in tuberculous lymphadenitis patients after transdermal delivery by EP. Possible explanations for these differences are as follows: firstly, although the skin sensitivity of guinea pig is similar to that of human being for cosmetic tests, it has a certain differences with human skin structure; Secondly, the skin structure of tuberculous lymphadenitis patients changed a lot, liquidation, thinning, ulceration happen frequently; thirdly, sampling depth in local skin of guinea pigs was different from that of pus in lesion of tuberculous lymphadenitis patients.

## Conclusions

Our present study showed that the application of EP significantly enhances INH penetration through skin in guinea pigs, while RIF patch alone can obtain therapeutic concentration in local skin. The fact that those concentrations were sustained above the MIC implied the efficacy of the drug delivery approaches to act on local tuberculosis. Further studies are required to address the best frequency and intensity of ultrasonic wave and electroporation for the transdermal delivery of commonly used anti-TB drugs.

## References

[CIT0001] Chen S, Qin M, Han Y, et al. (2016). Assessment of the efficacy of drug transdermal delivery by electro-phonophoresis in treating tuberculous lymphadenitis. Drug Deliv 23:1588–93 26669820 10.3109/10717544.2015.1124474

[CIT0002] Kost J, Pliquett U, Mitragotri S, et al. (1996). Synergistic effect of electric field and ultrasound on transdermal transport. Pharm Res 13:633–8 8710759 10.1023/a:1016070710397

[CIT0003] Maggi N, Pasqualucci CR, Ballotta R, Sensi P. (1966). Rifampicin: a new orally active rifamycin. Chemotherapy 11:285–92 5958716 10.1159/000220462

[CIT0004] Paudel KS, Milewski M, Swadley CL, et al. (2010). Challenges and opportunities in dermal/transdermal delivery. Ther Deliv 1:109–31 21132122 10.4155/tde.10.16 PMC2995530

[CIT0005] Polat BE, Hart D, Langer R, Blankschtein D. (2011). Ultrasound-mediated transdermal drug delivery: mechanisms, scope, and emerging trends. J Control Release 152:330–48 21238514 10.1016/j.jconrel.2011.01.006 PMC3436072

[CIT0006] Prausnitz MR, Bose VG, Langer R, Weaver JC. (1993). Electroporation of mammalian skin: a mechanism to enhance transdermal drug delivery. Proc Natl Acad Sci USA 90:10504–8 8248137 10.1073/pnas.90.22.10504 PMC47805

[CIT0007] Prausnitz MR, Langer R. (2008). Transdermal drug delivery. Nat Biotechnol 26:1261–8 18997767 10.1038/nbt.1504 PMC2700785

[CIT0008] Prausnitz MR, Mitragotri S, Langer R. (2004). Current status and future potential of transdermal drug delivery. Nat Rev Drug Discov 3:115–24 15040576 10.1038/nrd1304

[CIT0009] Ueda H, Sugibayashi K, Morimoto Y. (1995). Skin penetration-enhancing effect of drugs by phonophoresis. J Control Release 37:291–7

[CIT0010] Wiedersberg S, Guy RH. (2014). Transdermal drug delivery: 30+ years of war and still fighting. J Control Release 190:150–6 24852092 10.1016/j.jconrel.2014.05.022

[CIT0011] Zorec B, Becker S, Rebersek M, et al. (2013). Skin electroporation for transdermal drug delivery: the influence of the order of different square wave electric pulses. Int J Pharm 457:214–23 24076397 10.1016/j.ijpharm.2013.09.020

